# Rapid DNA detection of *Mycobacterium tuberculosis*-towards single cell sensitivity in point-of-care diagnosis

**DOI:** 10.1038/srep15027

**Published:** 2015-10-13

**Authors:** Benjamin Y.C. Ng, Eugene J.H. Wee, Nicholas P. West, Matt Trau

**Affiliations:** 1 Centre for Personalized NanoMedicine, Australian Institute for Bioengineering and Nanotechnology, The University of Queensland, QLD 4072, Australia; 2 School of Chemistry and Molecular Biosciences, The University of Queensland, QLD 4072, Australia

## Abstract

Although there have been many recent advances in Tuberculosis (TB) detection technologies, there still remains a major need to develop simpler point-of-care techniques. In an effort towards such a diagnostic test for resource-poor settings, we have designed a bioassay based on detecting amplified DNA via bridging flocculation. The assay is cheap, with a sensitivity approaching a single cell of *Mycobacterium tuberculosis* and the potential for translation into broader applications.

Tuberculosis (TB) is caused by the acid-fast bacillus *Mycobacterium tuberculosis* (*Mtb*) and has one of the highest infection rates in the world at 1 in 3 individuals, and 1.5 million deaths due to the disease in 2013 alone^1^. The World Health Organization (WHO) has identified several key factors for a successful global plan to stop TB. Among these factors is the urgent need to rapidly and accurately identify cases of TB so as to sharply reduce time to treatment and subsequently prevent disease transmission^1^. Conventional TB diagnostics include the tuberculin skin test and sputum smear microscopy, methods that are acknowledged to have several issues ranging from poor sensitivity or specificity and false negatives^2^,^3^,^4^. A relatively new test, the interferon-γ release assay, has shown promise with its sensitivity, but does not differentiate between active TB disease and latent TB infection^2^,^5^. Moreover, it requires storage of whole blood or viable white blood cells, which can pose a challenge in resource-poor settings^2^,^5^

While *Mtb* culture remains the gold standard for definitive diagnosis, it is a lengthy process that significantly delays time to treatment, and in turn, increases the risk of continued transmission of the disease and thus mortality rates^6^. Notably, people living in resource-poor settings are most direly affected by TB, mainly due to the ease of transmission as a result of poor living conditions. Another contributing factor is the greater population of HIV carriers in some of these regions, who are at an increased risk of developing active tuberculosis disease from *Mtb* infection^7^.

In resource-poor settings, the largest hurdle that remains is the difficulty in bringing the capability of detecting TB out of the clinic and into point-of-care (POC) – especially in areas where rapid turnaround diagnosis is most needed, and where sophisticated diagnostic equipment are unavailable^6^. In 2010, the Xpert MTB/RIF real-time PCR platform was endorsed by the WHO for the simultaneous diagnosis of TB and Rifampicin (RIF) resistance^1^,^8^. Most significantly, it is seen as an advance in the right direction of decreasing time to an accurate diagnosis^9^. However, the Xpert MTB/RIF device does present several limitations related to potential POC use. The high cost of instrumentation coupled with single-use cartridges impacts significantly on the cost of diagnosis, even after heavy subsidization through sponsorships^10^. Its reliance on a stable power supply, regular maintenance and calibrations represent a challenge in many remote settings^10^,^11^. Moreover, such platforms have limited throughput, which presents a potential problem in regions of high TB burden. Therefore, there is still a need for new diagnostic tools that can be rapidly and easily deployed in remote areas of high TB burden, and at a lower cost than conventional diagnostic methods^3^,^6^.

In recent years, methods to isothermally amplify DNA have become increasingly popular due to their potential of being adapted for use in DNA detection strategies out in the field. Unlike traditional PCR based methods such as that used in the Xpert MTB/RIF device, isothermal amplification methods circumvent the need for thermal cycling^10^,^12^. This is a major advantage when considering that a consistent supply of power for thermal cycling cannot be taken for granted in POC, especially at remote settings. Bioassays using an isothermal amplification method would have the important advantage of not being limited for use in settings with a dependable power source, as the reactions can be supported with a simple battery operated heat pack running at a constant temperature. Recently, body heat was shown to be sufficient to enable isothermal amplification^13^.

Of the various isothermal amplification methods, Recombinase Polymerase Amplification (RPA)^14^ was recently demonstrated for *Mtb* detection^15^. While useful, critical steps such as simple on-site sample processing and result interpretation still needs to be addressed in order to realize a working diagnostic bioassay suitable for POC applications. We have built on RPA to produce a more affordable, faster and simpler approach to detecting TB in POC.

Herein, we describe a method to interpret amplified *Mtb* DNA using bridging flocculation (Fig. 1). This bioassay is highly specific to *Mtb*, has a sensitivity of as low as 10 CFU, and does not require the use of expensive laboratory equipment or devices, making it an ideal candidate for development into a POC diagnostic assay in the near future. The phenomenon of bridging flocculation is typically used in water treatment systems to remove unwanted components, and has been recently repurposed for DNA detection^16^. The basic principle of bridging flocculation involves the use of a polymer chain long enough to bridge together particles into an aggregate under a specific buffer condition^17^,^18^,^19^,^20^. A critical aspect of bridging flocculation is the sudden shift between solution and flocculation phases which mirrors a binary situation/outcome, e.g. infected with TB or not. Another key feature which sets bridging flocculation apart from other commonly used nanoparticle-based DNA readouts^21^,^22^,^23^ is that it does not make use of tags or modifications to the DNA and/or particle or equipment to confirm the presence of DNA amplification – its simple reliance on the abundance of long amplified DNA segments for flocculation increases its value as a naked-eye, label-free, low cost bioassay. Therefore, a bridging flocculation-based assay may be useful for field detection of *Mtb*.

In this report, we describe in detail the application of a bridging flocculation assay to the detection of *Mtb* by establishing the level of sensitivity and specificity that may be useful for onsite *Mtb* detection. We have also described the DNA length requirements for bridging flocculation to take place which in turn, contributes new insights into the mechanism of this new DNA detection scheme.

## Results

### Determining the minimal DNA length for flocculation

While a bridging flocculation assay was described as a general nucleic acid detection platform^16^, the length of amplified DNA amplicons and the amount needed to trigger a flocculate has not been elucidated. A detailed characterization of the length and amount of amplicons will facilitate future assay design. To this end, DNA fragments of sizes 100 base pairs (bp), 200 bp and 300 bp were obtained using PCR and diluted with water to varying amounts of DNA. The bridging flocculation assay was then performed on these samples. As seen in Fig. 2, bridging flocculation occurred most robustly with products of at least 200 bases in length or more, and this phenomenon was triggered with as little as 25 ng of amplified DNA.

### Analysis of assay sensitivity

To determine the sensitivity, i.e, the minimum *Mtb* loading needed for a positive test response, we designed a set of primers that amplified a region within the RNA polymerase beta subunit (RpoB) gene of *Mtb*. Mutations occurring in a defined 81 base pair region within the RpoB, commonly termed the Rifampicin resistance determining region (RRDR), limit the effectiveness of Rifampicin as a drug^24^,^25^.

Using the primer set for the RRDR, we determined the limit of detection of this assay by titrating aliquots of *Mtb* cells and assaying them with our method. The same aliquots were also plated to estimate bacterial titre which was measured by the number of colony forming units (CFU). Gel electrophoresis was used to visualize the results of RPA amplification, and also to verify the fidelity of the bridging flocculation readout (Fig. 3A). Results showed that as little as 10 CFU could be detected with bridging flocculation. Even though a faint band corresponding to a dilution of 1 CFU was observed under gel electrophoresis, it was not sufficient for distinctive flocculation readout.

To examine the robustness of the assay in more complex solutions, 1 ng of human DNA was spiked into the RPA reaction (Fig. 3B). The increased template complexity had no effect on assay readout. Based on the whole genome sequence analysis of *Mtb* (Accession# CP000611)^26^, it was estimated that one genome equivalent of *Mtb* was approximately 5 fg of DNA. In the most dilute sample of 1 CFU, the amount of human DNA was approximately 200 000 times more than available *Mtb* template for amplification. Even so, similar sensitivity were obtained with the spiked experiments, with 1 CFU faintly detectable on gel electrophoresis and 10 CFU visually confirmed though bridging flocculation, thus further demonstrating the exquisite sensitivity and specificity of our assay.

### Specificity of assay

Previously, isothermal amplification of *Mtb* was performed utilising insertion elements IS6110 and IS1081^15^, presumably due to the multi-copy number of these elements in the *Mtb* genome and thus enhanced sensitivity. In the interest of demonstrating assay specificity towards *Mtb*, we designed a second set of primers that can be used in conjunction with the first set. These primers target regions specific to the *Mycobacterium tuberculosis* complex (MTBC) organisms, i.e. mycobacterium species that are known to cause TB. These regions, namely, the coding sequences of the early secretory antigenic target-6 (ESAT-6) and culture filtrate protein-10 (CFP-10), are important virulence determinants^2^,^4^. Using primers that target the above regions, we performed our assay on *Mtb*; *Mycobacterium smegmatis*, non-MTBC; and *Escherichia coli* (Fig. 4). 1 ng of gDNA for each bacterial species was used as template for RPA. Only *Mtb* tested positive by our assay, thus showing that a highly specific MTB assay can be designed with these choices of primers.

## Discussion

**Figure 1:**
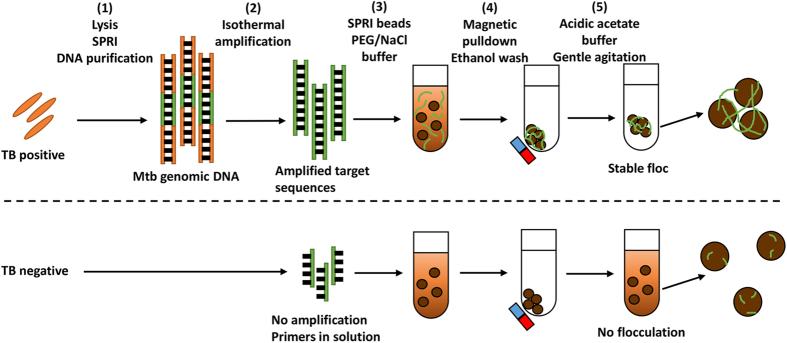
Schematic representation of the bridging flocculation strategy from DNA extraction to readout. Flocculation only occurs when there are sufficiently long chain DNA polymers, a result of the successful amplification of target sequences that indicate the presence of *Mtb*.

The results of this proof-of-concept assay showed that very small starting amounts of *Mtb* genomic DNA (gDNA) can be extracted and purified directly from culture using a modified Solid Phase Reversible Immobilization (SPRI)^27^,^28^ protocol with an optimized Guanidium-HCl lysis buffer (Fig. 1 and Methods), and subsequently amplified isothermally and detected with bridging flocculation. This assay does not rely on multiple centrifugation steps often used in conventional DNA extraction and is thus more suitable for POC applications.

**Figure 2:**
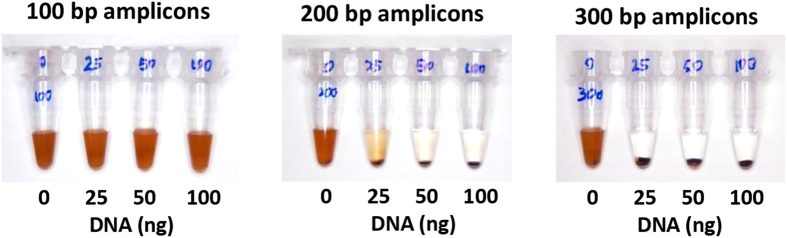
Minimum DNA size and amount for flocculation. A minimum DNA polymer size of 200 bp is required to trigger a visually distinctive flocculation result. This happens even at 25 ng of DNA product post amplification. Each figure is representative of at least 3 experimental replicates.

In the interest of furthering the understanding of the DNA-mediated bridging flocculation readout, we have also examined the minimum length of amplicons (200bp) needed to enable a robust assay (Fig. 2). The DNA amplicon cut-off size in the literature for a SPRI purification is 100–200bp^27^,^28^. Since the flocculation assay also used an SPRI-like approach to first load DNA onto beads, our data was consistent with the literature. This also suggested that one may be able to adjust the PEG/NaCl binding buffer conditions to improve assay specificity by selecting for a specific size of amplicon, thus underscoring the flexibility of the flocculation assay for isothermal methods that are prone to non-specific low molecular weight amplicons.

Although isothermal amplification of *Mtb* DNA has been reported in the past^15^,^29^, our work seeks to further improve on the method by incorporating a suitable DNA purification process and a DNA readout technology that integrates into a complete bioassay amenable to point-of-care diagnosis. In addition, this present study focuses entirely on TB detection and is the first report to establish the levels of sensitivity and specificity, and thus feasibility of a flocculation assay for TB diagnostics.

**Figure 3:**
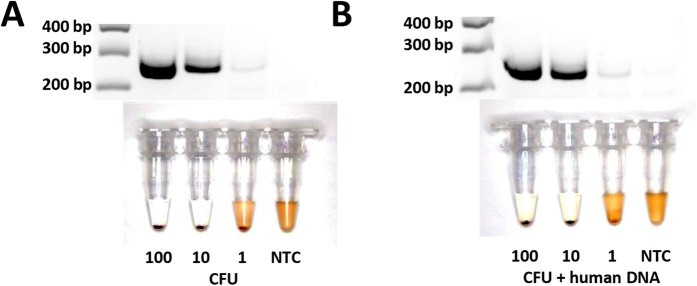
Sensitivity of assay in detecting *Mtb.* (**A**) Limit of detection of *Mtb* serially diluted 10-fold (L-R) 100, 10, 1 CFU and no template control. (**B**) Repeat of experiments in Fig. 3(A) with 1 ng of human DNA spiked into RPA. Each figure is representative of at least 3 experimental replicates.

In our sensitivity study, results pointed to a sensitivity of 1 CFU of starting bacterial titre detectable on gel electrophoresis, and 10 CFUs with bridging flocculation (Fig. 3). This level of sensitivity result was consistent even when a relatively large amount of background human DNA was spiked into the reaction before amplification, and this background DNA did not contribute to flocculation. The lack of flocculation in human DNA controls demonstrated that the assay was only sensitive to very high levels of DNA such as that produced after an amplification reaction – a necessary control to avoid false positives. While 1 CFU worth of starting DNA material was enough for positive readout on gel electrophoresis, a minimum of 10 CFUs was needed to trigger a visually distinctive flocculation result (Fig. 3). This was further evidence alluding to a minimum threshold of both length and amount of DNA product needed before flocculation.

We believe that an assay possessing high sensitivity is warranted in circumstances where samples contain very low amounts of *Mtb* DNA, such as that presented in childhood TB^30^,^31^. The paucibacillary nature of childhood TB creates several issues for conventional diagnostic tests routinely used for adults, e.g. lengthened the time to confirmatory diagnosis for culture and sharp reduction in the sensitivity of smear microscopy^31^. In a recent study, it was shown that trace amounts of *Mtb* DNA can be sampled and detected via PCR from the oral mucosa of adult TB patients due to the passing of bacilli from the airway through the mouth^32^. This can potentially be the case for childhood TB, and if true, can be an effective sampling technique for downstream detection with our bridging flocculation assay.

**Figure 4:**
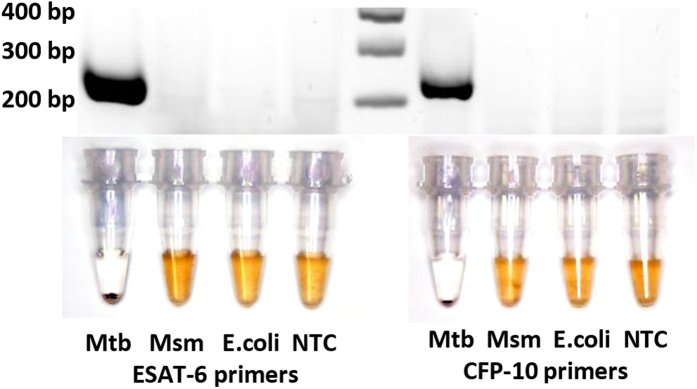
Specificity of assay. Primers targeting ESAT-6 and CFP-10 with templates *Mtb*, *Mycobacterium smegmatis* (Msm) and *Escherichia coli* (E.coli). Each figure is representative of at least 3 experimental replicates.

The results of our specificity study also show that our assay can avoid potential issues with cross-reactivity between mycobacterial strains by targeting highly *Mtb*-specific sequences present in the coding sequences for ESAT-6 and CFP-10 (Fig. 4)^2^,^4^. ESAT-6 and CFP-10 are proteins routinely used in interferon-γ release assays for *Mtb* detection for their superior specificity^2^,^4^. The use of ESAT-6 and CFP-10 in DNA-based detection assays may be better alternatives to the commonly used IS6110 and IS1081 insertion elements^15^ which are shared by many mycobacterial species^33^.

Based on these results, we have also observed that our naked-eye, discrete yes/no assay is in line with requirements for a point of care diagnostic test as laid out by accepted international standards^34^,^35^. We have developed a low cost (~USD $2) yet sensitive (1–10 CFU) assay platform that takes less than 90 minutes in total (from sampling to readout) for the detection of *Mtb* in POC settings via a bridging flocculation assay. Notable POC requirements include the need for a diagnostic test to (1) produce definitive yes or no results (2) within 3 hours, and (3) a minimal reliance on equipment, devices or professional training. A simple test with these characteristics may result in more TB infected people receiving timely and appropriate treatment as compared to the best laboratory based assays, significantly reducing the burden of the disease^35^.

As an extension, this assay might also prove beneficial for amplification systems that yield small amounts of non-specific amplification or primer-dimer artefacts, as these products would not be sufficiently long or in adequate amounts to trigger a bridging flocculation. Nevertheless, good primer design that yield minimal non-specific amplification is still important for good assay performance.

In conclusion, we believe that our assay has promise in the area of preliminary screening for TB in national healthcare systems, especially in resource-poor settings. We envision our assay could bridge the gap by complementing current diagnostic methods as a quick, sensitive and low-cost screening assay for pre-selecting patients in need of urgent medical intervention while confirmatory TB diagnosis are processed by slower and more expensive conventional methods.

## Methods

### DNA sample acquisition

*M. tuberculosis* H37Ra was cultured in Middlebrook 7H9 complete broth with supplementation (10% ADC, 0.2% glycerol and 0.02% tyloxapol). Samples for assay testing were taken at mid-exponential phase (OD_600_  =  0.8). These samples were serially diluted and plated on Middlebrook 7H11 agar (0.5% glycerol and 10% OADC) and the number of colony forming units (CFU) was visually determined.

### DNA extraction and purification

*M. tuberculosis* genomic DNA was extracted directly from culture by adding 2 volumes of an optimized lysis buffer (100 mM Tris-HCl pH 8.0, 3 M guanidium-HCl. 400 ng/μL RNase A and 2% v/v Triton-X) to 1 volume of culture. After 15 min incubation at room temperature, DNA was purified with SPRI. 1 volume of lysate was incubated with 2 volumes of 1 micron carboxyl coated magnetic beads (Thermo Fisher, Cat# 4515-2105-050250) in a binding buffer (10 mM Tris-HCl pH 8.0, 20% PEG8000, 2.5 M NaCl) for 10 min. These DNA bound beads were then separated from the lysate with a magnet and washed twice with 100% isopropanol, twice with 80% ethanol and eluted in one volume of water. All chemicals were purchased from Sigma Aldrich.

### Nucleic acid amplification

Nucleic acid amplification was performed with the TwistAmp Basic RPA Kit (TwistDX, cat# TABAS01kit) as recommended by the manufacturer with some modifications. 12.5 μL reactions were performed at 38 °C for 20 min using 1 μL of the nucleic acid extraction and 800 nM of each primer (Table 1). Following amplification, 3 μL of the RPA reaction was visualized by gel electrophoresis.

### Bridging flocculation assay

Two volumes of SPRI bead solution was added to 5 uL of amplified product and incubated for 5 min. After bead separation with a magnet and an 80% ethanol wash, 50 μL of flocculation buffer (100mM sodium acetate, pH 4.4, 1% v/v Tween20) was added to the beads and left to stand for 1 minute. The beads are then gently agitated until beads in the no template control are fully dispersed.

## Additional Information

**How to cite this article**: Ng, B. Y. C. *et al.* Rapid DNA detection of *Mycobacterium tuberculosis* - towards single cell sensitivity in point-of-care diagnosis. *Sci. Rep.* **5**, 15027; doi: 10.1038/srep15027 (2015).

## Figures and Tables

**Table 1:** List of RPA primers used. GenBank Accession numbers are as given.

Target/GenBank Accession	5′-Forward-3′	5′-Reverse-3′
*M. tuberculosis* RRDR CP003248.2	AACCGACGACATCGACCACTTCGGCAACCG	CCAGCGCCGACAGTCGGCGCTTGTGGGTCAA
*M. tuberculosis* ESAT-6 CP003248.2	CAATCCAGGGAAATGTCACGTCCATTCATTCC	CCTATGCGAACATCCCAGTGACGTTGCCTTC
*M. tuberculosis* CFP-10 CP003248.2	ATTTTGGCGAGGAAGGTAAAGAGAGAAAGTAGT	GAGTTCCTGCTTCTGCTTATTGGCTGCTTCTT

All primers were purchased from Integrated DNA Technologies (IDT).
